# Factors influencing the use of video interpretation compared to in-person interpretation in hospitals: a qualitative study

**DOI:** 10.1186/s12913-020-05720-6

**Published:** 2020-09-11

**Authors:** Eli Feiring, Stine Westdahl

**Affiliations:** Department of Health Management and Health Economics, University of Oslo, PO Box 1089, Blindern, 0317 Oslo, Norway

**Keywords:** Interpretation services, Hospitals, Norway, Austria, Qualitative, Migrants, Equity

## Abstract

**Background:**

Facilitating access to professional interpretation services is key to equitable hospital care for migrants with limited language proficiency; however, interpreter underuse has been documented. The factors that potentially enable or hinder professional interpreter use are not well understood. We aimed to compare perceptions held by hospital managers and healthcare practitioners of the factors influencing the use of remote video interpretation and in-person interpretation.

**Methods:**

This study employed a retrospective qualitative design. Two hospitals, located in Austria and Norway, with adequately similar baseline characteristics were purposively selected. Both hospitals used in-person interpreters, and the Austrian hospital had recently introduced remote video interpretation as an alternative and supplement. Fifteen managers and healthcare practitioners participated in focus groups and individual interviews. Data were thematically analysed with the aid of behavioural system theory.

**Results:**

Across sites, the facilitators of interpreter use included individual factors (knowledge about interpreter services, skills to assess when/how to use an interpreter, beliefs about favourable consequences), as well as organisational factors (soft budget constraints). Barriers were identified at the individual level (lack of interpersonal skills to handle difficult provider-interpreter situations, lack of skills to persuade patients to accept interpreter use, lack of trust in service professionalism), and at the organisational level (limited interpreter availability, time constraints). The introduction of remote video interpretation services seemed to counteract the organisational barriers. Video interpretation was further perceived to enable patient confidentiality, which was regarded as a facilitator. However, video interpretation introduced specific barriers, including perceived communication deficiencies.

**Conclusion:**

This study has identified a range of factors that are perceived to influence the use of interpreters in hospitals. The research suggests that-implementing remote video interpretation services lessens the barriers to use and that such services should be introduced in hospital settings as an alternative or supplement to in-person interpreters. Further intervention functions should be considered to bring about change in the use of interpretation services, including developing guidelines for interpreter use, educating staff in the appropriate use of video technology, and training staff in communicating with interpreter and patients with limited language proficiency.

## Background

Migration is increasing across the Western countries, and many people are unable to interact with the healthcare system in the country in which they live because of limited language proficiency (LLP) [1, 2]. Language discordance create what has been described as a “triple threat” to effective communication, as language differences not only hinder interaction but also act as a barrier due to their association with culture and often with low health literacy [3, 4]. A growing body of research has documented a range of adverse effects of language discordance on quality of care, health outcomes, patient satisfaction, and costs [5–9]. Studies indicate that linguistic barriers result in inappropriate diagnoses, poorer adherence to treatment and follow-ups, more medication complications, more and often unnecessary diagnostic tests being ordered, and longer hospitalisations [10, 11].

To ensure equity in health and healthcare for all patient regardless of cultural and linguistic barriers, migrant friendly hospital initiatives have been identified as a priority in many European countries as well as in Australia, the United States (US), Canada, and Israel [12]. These initiatives include facilitating access to professional interpretation services. Despite evidence of the benefits of using such services [6, 8, 9, 13–15], professional interpreter underuse has been documented [16–18]. Two main barriers to use have been identified: time pressure and limited interpreter availability [18].

As new technologies, such as telephonic and remote video interpretation services, are developed and implemented, accessibility to professional interpreters improves [2]. Nonetheless, previous studies suggest that even when professional interpreters are available, services are underused [19–21]. This is unfortunate. The specific factors that potentially enable or hinder professional interpreter use are not well understood. Diamond et al. reported that doctors at US hospitals found it easier to” get by” without an interpreter and cited both individual and practice environmental factors underlying doctors’ decisions about interpreter use [18]. Parsons et al. studied Canadian doctors’ experiences of care provision in situations of language discordance and concluded that a doctor’s decision to “get by” versus “get help” from an interpreter rested on the judgment of whether communication was considered “good enough” [20]. Both studies were conducted in settings in which where English was the dominant language. There is, however, a paucity of research eliciting health practitioners’ views on this topic in the European context and especially, in contexts in which English is not the main language, which might make the language barrier more profound (but see [17, 22]). Remote video interpretation offers the promise of better access to a range of languages; however, little is known about the factors influencing the use of remote video interpreters. To address this gap, the present study sought to yield information across contexts about the factors perceived by managers and healthcare practitioners to determine the use of professional interpretation services in hospital settings in which English was not the dominant language and to compare remote video interpretation services to those provided in person. This paper presents the findings of the study and aims to (1) provide an in-depth examination of the perceived determinants of the use of interpreters across contexts, (2) compare perceptions of the factors influencing the use of video interpretation and in-person interpretation to identify whether the implementation of video interpretation changes the perceptions of the determinants of interpreter use, and (3) consider implications for health services and health policy.

## Methods

The study employed a quasi-experimental qualitative design [23]. We purposively (non-randomly) selected two hospitals located in Austria (Hospital A) and Norway (Hospital B). Both hospitals were large university hospitals in urban settings, providing for multilingual patient populations including migrants with limited proficiency in the countries’ official languages. Both hospitals used in-person interpreters and telephonic interpretation services. Furthermore, in 2015, the Austrian hospital implemented remote video interpretation services as a supplement and alternative to in-person interpreters. Thus, in the present study, Hospital A represented the intervention case (using video interpretation), while Hospital B represented the control case (not using video interpretation). Table 1 gives a short overview of some characteristics of the study setting. The COREQ checklist was used to report the study (Additional file).

**Table 1:** Characteristics of study setting [24–27]

	Austria	Norway
Population size	8.77 mil.	5.29 mil.
Immigrants	15.3% (mostly European countries, and Asia)	14.1% (mostly European countries, and Asia)
English as official language	No	No
Population diversity reflected in workforce of healthcare system	No	No
Patient’s right to information about care	Yes	Yes
Healthcare personnel responsible for providing professional interpreters	Yes	Yes
Public/private financing of interpretation services	In-patient care: majority public, free at the point of access Patients not attending scheduled visit with interpreter must pay a fine	In-patient care: public, free at the point of access
National guidelines about when/how to use interpreters	No	No
Specific regulations apply to refugees /asylum seekers: entitlements restricted to basic healthcare	Yes	Yes

### Sampling and recruitment

Purposive sampling of study participants was conducted. We wanted to interview staff who had experience with the use of interpretation services, such as managers, clinicians, and personnel who were responsible for ordering interpretation services (i.e. nurses at Hospital A, medical secretaries at Hospital B). The study benefited from a pre-existing relationship with both hospitals (as one of the authors (SW) had been a student intern). Three clinics that used interpretation services frequently were approached at each hospital. We were helped by the clinics’ management to identify potential participants who had knowledge of the ordering and use of interpretation services and of English/Norwegian and, further, to distribute written information about the study. A total of 15 participants were recruited for the study and provided informed written consent to participate.

There are no commonly accepted standards of determining adequate sample size in qualitative research [28]. It has been suggested that at least 10 interviews should be conducted initially, followed by further data-gathering until no more important new information is obtained [29]. We aimed at thematic saturation [30], that is, the extent to which the categories of a predetermined framework are adequately represented in the data. We decided that thematic saturation was sufficiently achieved in this study when three focus groups and four individual interviews had been conducted.

### Data collection

The data were collected between March and May 2018. Initially, we planned to arrange four focus groups: one with staff and one with managers at each hospital. The focus groups would enable us to explore the complexity surrounding the use of interpreters and encourage participants to discuss the topic with other participants [31]. Three focus groups (2 to 6 persons, 40 to 60 min) were conducted; however, due to the participants’ busy schedule and time limits we pragmatically decided to interview the last four participants individually. These interviews lasted 30 to 40 min.

The data collection was conducted by one of the authors, SW, under the supervision of EF. The focus group and interviews at Hospital A were conducted in English, while the focus groups at Hospital B were conducted in Norwegian. The data collection took place at the participants’ workplaces and was guided by semi-structured topic guides (one guide for managers and one for healthcare practitioners) that explored six main topics: perceptions of accessibility of interpretation services, perceived benefits and challenges of using interpreters, views about the resources needed, previous experiences with the use of interpreters, perceptions regarding patient views and the appropriateness of using interpreters, and the manager’s role regarding the provision and organisation of interpretation services (see Additional file). The guides were developed by the authors and discussed with a representative from each hospital to improve relevance and clarity.

All the focus group and interview data were audio-recorded and transcribed verbatim. The participants were given the possibility to read copies of de-identified data transcripts and provide feedback. The data were stored electronically on a password-protected computer.

### Data analysis

**Fig. 1:**
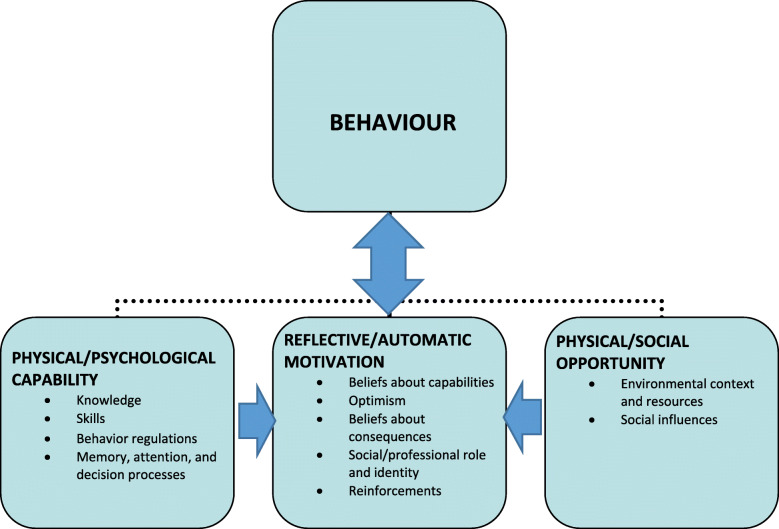
The COM-B system of behaviour, including TDF domains (adapted from [23, 33, 34])

The data content was identified based on a predetermined analytical framework. We utilised the COM-B system, a framework developed by Michie et al., to guide the understanding of the behaviour and develop behavioural targets as a basis for intervention design [32]. The framework proposes that three conditions are needed to perform Behaviour - namely, individual Capabilities (psychological and physical capacities to engage in the activity), organisational and social Opportunities (physical and cultural-social factors that lie outside the individual and make behaviour possible or prompt it) and Motivational factors (reflective and automatic processes that direct behaviour). The components can potentially influence each other in different ways. The framework is rather abstract. To unpack the COM-B further, we used the Theoretical Domains Framework (TDF) that has been conceptualised as an additional layer to the COM-B [32]. The TDF was initially developed as a synthesis that integrates constructs from behaviour change theories into 14 domains covering individual capabilities and motivation factors in addition to the organisational and social environment: knowledge; skills; behaviour regulation; memory, attention, and decision processes; beliefs about capabilities; role and identity; optimism; beliefs about consequences; reinforcements; intentions; goals; emotions; environmental context and resources; social influences [33, 34]. The domains provided subdivisions of the three main COM-B components, as illustrated in Fig. 1. Importantly, the framework does not propose testable relationships between elements and should, rather, be understood as a theoretical lens through which to view different influences on behaviour [34].

The two authors independently coded the data into the predetermined framework. The data were first coded and organised thematically in accordance with the COM-B categories. We then coded the sub-themes with the aid of the TDF with the aim of identifying factors that facilitate and/or hinder the use of interpreters. Coding was not always easy, as text units were at times ambiguous, were sometimes related to each other, and could be categorised under more than one sub-theme of the given framework. We discussed discrepancies until agreement. Further, each theme was compared between sites to produce an across-site understanding. We then selected compelling quotes to illustrate the themes, and both authors translated the Norwegian quotes into English for the purpose of presenting the results to an international audience.

## Results

**Fig. 2:**
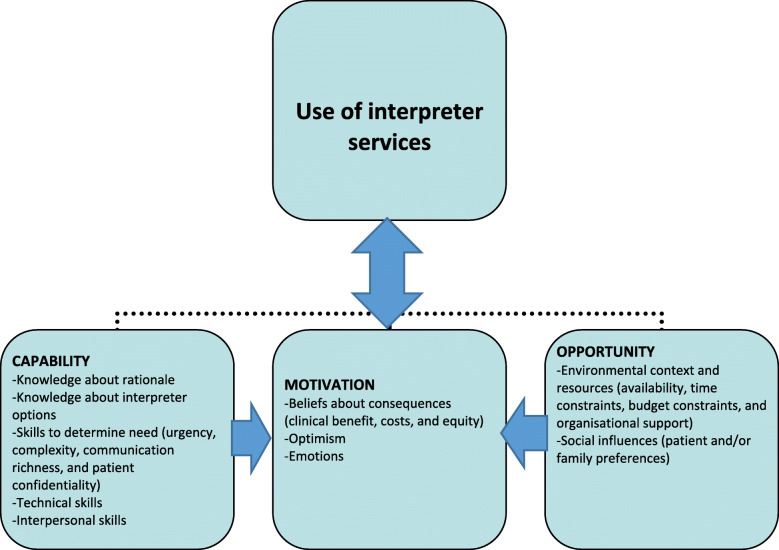
Determinants of interpreter use: summary of study results

A total of 15 participants (Hospital A (*n* = 6); Hospital B (*n* = 9)) were recruited for the study: managers (*n* = 4, all doctors), doctors (*n* = 2), nurses (*n* = 4), and medical secretaries (*n* = 5). The section below describes how the data align with the COM-B components and the TDF domains. The results are summarised in Fig. 2.

### Individual capabilities

Across the sites, participants identified individual capabilities, such as knowledge of interpretation services and the skills to adequately order and use the services, to be important determinants of the use of interpretation services.

### Knowledge

The participants reported that they endorsed the rationale of using interpretation services as a means to the efforts of giving all patients safe and high-quality care. The use of interpreters was regarded as a necessity in many situations.

*Patient safety is vital and poor communication often leads to poor treatment. To be able to provide the best quality treatment, the different people must understand each other.* (Medical secretary 4B)

*We would not be able to do what we need to do without interpreters, they are absolutely crucial.* (Manager 2B).

### Skills

The influences on the ordering and use of interpretation services included the skills to assess the needs to use interpreters.

*An interpreter should be used when it is clear that the patient doesn’t understand what is being said.* (Doctor 2A).

Further, the participants believed that it was essential to have skills to determine which type of interpretation service they should order: *It depends on (…*) *what kind of interpreter is needed* (Doctor 1A). Four different factors were important for assessment: situation urgency, situation complexity, the need for rich communication, and the need to protect the patient’s integrity and confidentiality. For example, the Hospital B participants reported that they preferred in-person to telephonic interpretation in situations that were expected to be complex in terms of their severity or the number of people involved, and further, in situations characterised by the need to address particular concerns of their patients. Telephonic interpretation was used only when there was an emergency or a check-up.

*We definitely use personal interpreters the most. It (the choice) relates to how the conversation conveys with the body language - what you read between the lines.* (Manager 1B).

Some of the Hospital A participants reported that video interpretation was preferred to in-person interpretation in situations in which clinical staff decided that there was a need for additional protection of the patients’ confidentiality. Video-interpretation provided a wider range of opportunities to use interpretation services that best suited the situation, because the staff could use the option to switch off the screen, thus having only the audio.

*If you have a patient that is a victim of violence, then it is better to use the video interpretation because it is more anonymous and has only the (individual’s) voice.* (Doctor 1A).

However, sometimes in-person interpretation was favoured because video interpretation was perceived as too detached to the situation.

*You lose personal contact*. (Nurse 2A).

*In sensitive situations (…*) *a personal interpreter understands the situation and does not ask unnecessary questions.* (Doctor 1A).

Other types of skills were also viewed as important to decision-making about the use of interpretation services. For example, technical skills were identified as a determinant of the use of video interpretation services among the Hospital A participants. Some reported that they were less familiar with and capable of using the technology and regarded this lack of skill to be a barrier to its use.

Further, across sites, the participants discussed how a lack of the necessary interpersonal skills to handle difficult situations with unprofessional interpreters or situations in which the patient or the patient’s family was unwilling to use an interpreter influenced the use of one. One participant explained how she and her colleagues used argumentative techniques to persuade the patient and felt that this could be challenging:

*There are situations when the patient doesn’t want an interpreter. In order to provide right care, we argue that the doctor needs an interpreter present”* (Medical secretary 2B).

### Individual motivation

Across the sites, the participants seemed highly motivated to use professional interpretation services due to their beliefs about the favourable consequences of doing so; however, at the same time, they reported other factors (including role conflicts and mistrust in interpreters) that had a negative influence on decisions about interpreter use.

### Beliefs about consequences

A range of outcomes was perceived to be gained from using interpretation services, including the avoidance of misunderstandings, reduced costs related to shorter hospital stays and less risk of readmission, all of which further motivated the service use.

*The quality of care and treatment gets better (…*) *because we avoid misunderstandings. The patient gets important information and we can reduce the length of stays (…*) *Using an interpreter makes our work easier* (Nurse 1B).

### Professional role and identity

In some situations, the patient or the patient’s family did not want an interpreter present. The participants perceived such situations as a conflict between patient autonomy and professional norms of providing safe and beneficial treatment. The medical staff felt that their role as providers responsible for appropriate treatment was challenged and wanted organisational support to back up their decisions about having an interpreter present. This was a situation that was well known to the managers, who reported that such support was given. One of them said the following:

*The doctor can override the patient and say, we have to have an interpreter present.* (Manager 2B).

### Emotions

Some participants at both sites voiced concerns about the quality of in-person interpreters. They spoke about experiences with “unprofessional” interpreters who were believed to lack language skills, did not arrive on time and interfered in the conversation. For example, some participants said that they felt uncomfortable because they did not trust the interpreters to be adequately knowledgeable regarding medicine and medical terminology. They also talked about situations in which the interpreter discussed topics that were irrelevant to the medical situation or did not seem to interpret all that was said, which again resulted in a lack of trust. In this way, the perceived lack of interpreter capabilities influenced participants’ motivation to use their services.

*Not all interpreters are certified in the specific medical terminology that is needed. I cannot be sure what is being said and I don’t know the quality of the interpretation (…*) *You don’t know the interpreter’s intention, you have to trust that they will be professional and will want to help.* (Doctor 1A).

However, compared to Hospital B, the motivation to use interpretation services seemed greater among Hospital A participants and seemed to evoke more positive emotions. The introduction of video interpretation was perceived as motivating.

*Now the system is working well. It is good always to have the opportunity to use the video interpretation. We are very satisfied. (*Nurse 3A).

### Organisational and social opportunities

A range of organisational and social opportunities was perceived as important determinants of the use of interpretation services, including interpreter availability, time, money, and the preferences of the patient and the patient’s family.

### Organisational resources

While monetary costs did not generate substantial discussions among the participants, they were clearly well aware of budgetary constraints. The costs per appointment were perceived as “high”, and the participants reported that the service should be used efficiently.

*We assess the situation and only order an interpreter if we really have to.* (Medical secretary 1B).

*The video interpretation is charged per 15 min, thus being well prepared and organised is essential*. (Doctor 1A).

However, the participants seemed to consider budget constraints as being “soft”.

*Managers are concerned about the costs (…*) *but they also want the patient and the doctor to understand each other.* (Nurse 1B).

*If we need it (the interpretation service), we use it.* (Nurse 3A).

The managers, in contrast, reported that the costs of interpretation services should be assessed relative to the risk of longer hospital stays and readmissions:

*It is an expensive service, but if you risk the patient having to come in again, it also costs money. I will not say, No. Let us skip the interpreter because it’ s so expensive”.* (Manager 2B).

Across the sites, the participants had undergone experiences with personal interpreters who had not always been available because of the limited opening hours of the services, long travel distances, and delays. Further, there was sometimes a need for the interpretation of less-used languages, and it was difficult to find someone to interpret these. In addition, some interpreters were difficult to reach:

*The interpreters do not always pick up the phone or (they) cannot come in.* (Nurse 3A).

Some participants said that ordering interpretation services was sometimes time-consuming, and creating extra work. Further, some participants found that it could be difficult to plan the need for interpretation services.

*It can be quite an effort to get an interpreter (…*) *We usually need the interpreter right away, but the appointment needs to be scheduled in advance.* (Nurse 1B).

Across the sites, the participants reported that they were reluctant to order professional interpreters for routine clinical interactions and instead tried to “get by” or “manage” without an interpreter or with the help of family, friends or bilingual staff. They further reported that although they knew that family members should not be used as interpreters, this was found to be the only alternative in emergency situations.

*Often, the adults don’t know Norwegian, but their children do and then, the children help to explain.* (Medical secretary 3B).

*Yes, especially in the emergency room, it’s important that family or a friend is with the patient and can help interpret*. (Nurse 3A).

However, the use of ad hoc interpreters was commonly described as “something that should not happen”. Several reasons were cited, including considerations of patient confidentiality, emotional ties, and lack of knowledge of medical terminology, as well as the negative consequences of these. One participants stated,

*When a family member interprets information, it’s often lost because the relative only interpret parts of the information.* (Manager 2B).

The availability of professional interpreters was however, perceived to have improved at Hospital A when video interpretation was introduced. The video interpretation service was accessible at all hours, both male and female interpreters were available, and the service offered a wide range of languages. The use of video interpretation was considered to lessen the practical work of ordering a personal interpreter. Further, the nurses considered it less “emotionally draining” to order a video interpreter because they were less dependent on one person’s availability and goodwill:

*It is a lot easier because we do not have to find somebody, call them and beg them to come in and help us.* (Nurse 3A).

### Social influences

Furthermore, social opportunities were identified across sites as a factor affecting the ordering and use of interpretation services. According to the participants, in some cases, the patient’s and/or the patient’s family preferences constrained the opportunity to use interpreters. This was a factor in terms of preferences for privacy and confidentiality.

*Some patients do not want an interpreter present; because they are afraid, it might be someone from their own community.* (Manager 2B).

## Discussion

To the best of our knowledge, this is the first study of the use of interpretation services that has applied a theoretically based framework to aid analysis. The combined COM-B/TDF framework proposes that individuals’ capabilities, motivation and organisational and social opportunities are needed to perform a behaviour [32]. One important insight from behaviour theory is that all behaviours occur within a system of other competing or contributing behaviours. In this study, we utilised the framework to investigate the use of interpreter services and provide an analysis of the determinants of the use that will possibly help to define what needs to be changed in order for interpretation services to be used more optimally.

Our findings revealed that a range of factors was perceived as influencing the use of interpretation services. Across the sites, the enablers included being knowledgeable about the services, having the skills to assess the need for interpreters and the most adequate type of interpretation services, and being motivated by expectations of the positive consequences of use. Further, organisational factors, such as soft budget constraints and managerial support for the use of interpretation services were identified as facilitators. Others have pointed out that health practitioners opt for “convenience” and “the path of least resistance” when making decisions about use of interpretation services [18, 20]. The present study shows that the participants recognised barriers to interpreter use at the individual and organisational level.

One important barrier involved the perceived lack of interpersonal skills to handle difficult situations. For example, dealing with patients/families who did not want professional interpreters present resulted in conflicts of interests between professional obligations and patient autonomy. This finding resembles that of Yelland et al. [19]. Further, as documented by others, experiences with interpreters who were described as unprofessional, resulted in mistrust and discouraged the use of interpreters [11, 22]. Such challenges can only be overcome if health practitioners are trained to handle dilemmas in their encounters with interpretation services and know the professional obligations of interpreters. Brisset et al. described how the presence of an interpreter may result in the health practitioner feeling a sense of loss of intimacy with the patient and that the practitioner may insist on the neutrality of the interpreter [22]. The interpreter may, in contrast, be expected to provide emotional support and cultural brokering, as documented by Kale et al. [17]. Our study suggests that health practitioners need to be more aware of the degree to which they are comfortable with the involvement of interpreters. Dilemmas related to the interpreter’s role may become more difficult due to time- and budgetary constraints: previous research indicates that patients are given a vast amount of information in a short period when an interpreter is present, because clinical staff prepare for “one big conversation”, rather than a few shorter sessions [10]. Further, the use of interpreters itself adds time to the consultation. There will hardly be time left for discussions about breach of professional detachment, impartiality or confidentiality.

Regulation of interpretation services is needed to promote adequate use of interpretation services in hospitals. Guidelines for the use of accredited interpretation services, including the documentation of language barriers in patients’ records and the criteria for ordering services, should be developed and implemented.

### Factors influencing use of remote video interpretation as compared to in-person interpretation

The introduction of remote video interpretation clearly counteracted some important barriers to the use of in-person interpreters. For example, the organisational-level factors that were perceived as important barriers to the use of in-person interpreters at Hospital B, such as time constraints and a lack of interpreter availability, were less frequently referred to as barriers among the Hospital A-participants who had the possibility of using remote video interpretation.

Furthermore, while patient preferences for anonymity were identified as a barrier to the use of in-person interpretation, Hospital A-participants acknowledged the option to enable patient privacy and confidentiality by turning off visual communication when using video interpretation. This option to use the service in a flexible manner that best fit the situation was regarded as motivating interpreter use.

The implementation of video interpretation was, however, perceived to have introduced specific barriers, such as a more detached and impersonal mode of communication, which was not always regarded as beneficial. The lack of technical skills was further perceived to hinder the use of video interpretation.

To bring about change in the context of the use of interpretation services, various intervention functions should be considered. Importantly, video interpretation offers availability and flexibility, thereby increasing access and possibly dis-incentivising decisions to “get by” without interpreters. The introduction of video technology should therefore be considered in hospital settings. Interventions such as training healthcare practitioners to work with interpreters and developing the skills to use technology such as the tablet/computer devices used in video interpretation, are available to target the specific barriers to the use of remote video interpreters. Table 2 summarises the comparison of in-person and video interpretation.

**Table 2:** Comparison of factors perceived to determine the use of interpretation services: Perceived enablers (+) and barriers (-).

		In-person interpretation	Video interpretation
Capability	Knowledge	+	+
	Skills to determine need	+	+
	Technical skills	+	–
	Interpersonal skills	–	–
Opportunity	Availability	–	+
	Time	–	+
	Costs	–	–
	Organisational support	+	+
	Patient/family preferences	–	+
Motivation	Beliefs about consequences	+	+
	Optimism	–	+
	Emotions	–	–

### Strengths and limitations

A strength of our study was the use of theory in a field that has largely taken an empirical approach to the exploration of barriers to interpreter use. This allowed us to give a detailed analysis of themes within the data and to provide a systematic identification of potentially modifiable factors that may affect the implementability and use of interpretation services. However, the use of a predetermined framework may also be a limitation of the study, as there is a risk of losing important data: text units that do not fit the framework and are thus left out of the analysis can be identified [35]. This may provide a less rich description of the data.

As recognised by the developers of the COM-B/TDF framework, coding can sometimes be difficult: text may seem to fit into multiple domains [34]. For example, some of the participants said that they felt uncomfortable because they did not trust the interpreters to be adequately knowledgeable about medical terminology. This finding could be coded as a lack of interpreter capability. We coded the text into the domain that best reflected the key theme - motivation - because the participants’ motivation to use interpreters was affected by their perception of the interpreters’ lack of capability.

We chose to compare the perceptions about interpretation services in two different countries. Undertaking a cross-country comparison of aspects of healthcare systems is always difficult, given the complexity of healthcare and the array of political, institutional, and cultural contexts. However, a comparison across countries may help to avoid false particularities (“everywhere is special”) and false universalism (“everywhere is the same”) and may contribute to cross-country learning [36, 37].

The hospital management helped us to identify potential participants. This was a pragmatic choice, given the time constraints. We cannot rule out the possibility that bias was introduced in the selection process. Further, four participants did not have time to participate in the scheduled focus groups. We pragmatically chose to interview those participants individually. We cannot rule out the possibility that some participants would have provided different data if subjected to a different data collection method.

The sample size was relatively small and is a limitation of the study. We observed variations in the use of interpretation services across the different clinics at each hospital. However, the sample was too small to investigate the variation. Further work on variation across medical specialities is required. In a similar vein, due to the small sample size, we were unable to compare variations in the perceptions of use between the different groups of health practitioners included in the study.

We did not include patients in this study. Previous research suggests that patients are indifferent regarding the choice between in-person or video interpretation [38]. However, as remote video interpreting raises challenges related to patient confidentiality and the protection of patient information, the perspectives of patients require further exploration [39].

## Conclusion

Utilising the combined COM-B/TDF framework, this study aimed to offer a theoretically informed analysis to improve hospitals’ responses to the needs of patients with LLP to understand and communicate with caregivers and thus contribute to improving the quality of care. The factors perceived to determine the use of interpretation services include health practitioner capability and motivation in addition to organisational and social opportunity. Video interpretation may counteract barriers to interpreter use, such as limited interpreter availability and time constraints, and can be facilitated by training staff in the appropriate use of the technology and interaction with interpreters. Guidelines for the use of accredited interpretation services should be developed and implemented.

## Supplementary information

**Additional file 1.**

## Data Availability

The data reported in the article (qualitative interview data) are available via a request made directly to the corresponding author.

## Abbreviations

COM-B: The Capability, Opportunity, and Motivation Behaviour system
TDF: The Theoretical Domains Framework
